# An Unusual Case of Hereditary Neuropathy With Liability to Pressure Palsy: A Diagnostic Challenge

**DOI:** 10.7759/cureus.33306

**Published:** 2023-01-03

**Authors:** Kumar Saurabh, Reyaz Ahmad

**Affiliations:** 1 Department of Neurology, Tata Main Hospital, Jamshedpur, IND

**Keywords:** multifocal acquired demyelinating sensory and motor neuropathy (madsam), chronic inflammatory demyelinating polyneuropathy (cidp), hereditary neuropathy with liability to pressure palsy (hnpp)), neuropathy, atypical cidp

## Abstract

Hereditary neuropathy with liability to pressure palsy (HNPP) is a genetic condition in which individuals develop recurrent nerve palsies due to nerve injury at susceptible anatomic sites. Because of its rarity, other diseases usually appear high in the differential list when the clinical presentation is suggestive. Here, we describe a case of HNPP initially thought of as radiculopathy and focal chronic inflammatory demyelinating polyneuropathy (CIDP). Only on close clinical examination, supportive electrodiagnostic tests, and recurrence with typical history, a diagnosis of HNPP was suspected and later confirmed by a genetic test.

## Introduction

Hereditary neuropathy with liability to pressure palsy (HNPP) is a genetic condition caused by 1.5 Mb deletions in the 17p11.2 chromosome locus involving the *PMP22* gene in 80% of cases. Point mutations in the same gene cause another 20% of cases. *PMP22* gene product (a transmembrane glycoprotein) is an essential component of myelin helping in the maintenance of its compact structure. Its deficiency leads to increased susceptibility of nerves to minor traumas. Another common hereditary neuropathy, Charcot-Marie-Tooth (CMT) disease type 1 is caused by duplication involving the same *PMP22* gene. HNPP is transmitted in an autosomal dominant pattern with variable expressivity. About 20% of cases are due to de novo mutation [1]. It has a prevalence of 7-16/100,000 population. The disease is also called tomaculous neuropathy because of the presence of tomacula in nerve biopsy findings. Tomacula are multifocal hypermyelinating lesion that appears longitudinally as a chain of sausage. These are characteristic of HNPP but can also be found in other diseases like CMT [2]. Nerve enlargement because of tomacula formation is seen most prominently at entrapment sites [3]. Patients typically present with neuropathy resulting from nerve compression at entrapment sites like ulnar neuropathy at the elbow, peroneal neuropathy at the fibular neck, carpal tunnel syndrome, radial nerve palsies, etc. Many times, diagnosis is not suspected first because of its rarity. Common differentials for focal neuropathy include both genetic and acquired conditions like CMT, non-hereditary common pressure palsies, vasculitic neuropathy, chronic inflammatory demyelinating polyneuropathy or CIDP (particularly focal CIDP and Multifocal Acquired Demyelinating Sensory and Motor Neuropathy or MADSAM variant), etc. [4]. Our patient initially presented with a left foot drop. He had a history of back pain radiating to the posterolateral aspect of the left thigh for one year. For the last six months, he also complained of heaviness of both lower limbs on walking about 50 m, which subsided on sitting for some time indicating spinal canal stenosis. MRI of the lumbosacral (LS) spine done six months back showed grade 1 anterolisthesis of L5 over S1 and asymmetric broad-based and focal posterior disc bulge at L5-S1 level indenting the thecal sac. X-ray of LS spine done just before surgery showed progression of anterolisthesis to grade 2. He was operated on for L5-S1 disc prolapse and L5-S1 anterolisthesis 15 days back with L5 laminectomy, L5-S1 discectomy, and L5-S1 fixation. Upon initial impression, it seemed that the patient developed foot drop due to worsening radiculopathy or some surgical complication. When symptoms progressed after two months, the patient was diagnosed as focal CIDP because of conduction block in nerve conduction studies, increased cerebrospinal fluid (CSF) protein, and improvement with steroid therapy. However, the patient again developed a right foot drop after two months, the precipitating event being prolonged crossed-leg sitting in the office. This typical history led to suspicion of HNPP, which was later confirmed by genetic analysis showing heterozygous deletion of the *PMP22* gene.

## Case presentation

A 43-year-old male presented in the outpatient department with a complaint of left foot drop. He was a known case of L5-S1 disc prolapse and L5-S1 anterolisthesis and was operated 15 days back with L5 laminectomy, L5-S1 discectomy, and L5-S1 fixation. There was an isolated weakness of left foot dorsiflexion (power- 3/5). All other muscle groups had 5/5 power. Plantar reflex was bilaterally down-going. There was no sensory or proprioceptive deficit. Both ankle and knee reflexes were absent bilaterally. Upper limb reflexes were within normal limits. He was advised nerve conduction study and electromyography (NCS/EMG) for lumbosacral plexus and asked to follow up with reports. However, the patient was lost to follow-up.

He again presented after two months with a complaint of worsening left foot drop. This time he was admitted for a workup. He was advised physiotherapy for his foot drop awaiting a specific diagnosis. There was no history of pain or trauma. He was a known diabetic and hypertensive for the last 10 years which are well controlled with medications. He takes a mixed diet and has no addictions. There is no history of similar illness in family members. On examination, there was no pallor, cyanosis, clubbing, icterus, or edema. Chest, cardiovascular, and abdominal examinations were within normal limits. Bulk and tone were normal in all limbs. There were no fasciculations. The power of the left foot dorsiflexor and evertors were 2/5 and 3/5, respectively. Rest all muscle groups had 5/5 power. Ankle and knee reflexes were bilaterally absent. Upper limb reflexes were within normal limits. There was a sensory deficit in the first dorsal webspace corresponding to deep peroneal nerve territory. Other neurological examinations were within normal limits.

He was advised NCS examination which showed the right peroneal nerve (EDB) having delayed latencies (9.21 ms) and reduced amplitudes (0.8 mv) and the left peroneal nerve (EDB) showing conduction block (ankle stimulation- 4.4 mv amplitude, below knee stimulation- 0.9 mv amplitude). Bilateral superficial peroneal nerve recordings were absent. Median nerve latencies were increased bilaterally. These findings fulfilled the electrodiagnostic criteria for MADSAM or possible focal CIDP. However as there was no history of weakness in the right lower limb, a diagnosis of possible focal CIDP was more appropriate according to revised European Federation of Neurological Societies (EFNS) criteria, 2021. EMG showed reduced recruitment with high amplitude, long duration polyphasic motor unit action potential (MUAP) in bilateral tibialis anterior and extensor digitorum brevis muscles indicating a more chronic process at work. CSF examination showed elevated protein (91.3 mg/dl), cells- six (all lymphocytes), and sugar- 81.3 mg/dl (corresponding blood sugar- 129 mg/dl). Findings were suggestive of albumin-cytological dissociation. Based on the history of stepwise or progressive weakness for two months, weakness and sensory deficit localized to left peroneal nerve distribution, decreased local reflexes (including knee reflex not explained by radiculopathy), and findings of conduction block in the left peroneal nerve at the non-entrapment site (between below knee and ankle in NCS examination) a diagnosis of possible focal CIDP was made and the patient was discharged on tablet prednisolone (40 mg/day). A lower dose of steroid was prescribed because of the risk of steroid-induced hyperglycemia in a diabetic patient. He was advised regular blood glucose monitoring at home. It was planned to modify steroid dose on the basis of disease progression and blood glucose levels. On follow-up for the next two months, patient showed gradual improvement in the weakness of left foot dorsiflexion and eversion (2/5 to 4/5 and 3/5 to 4/5, respectively). Improvement with steroid therapy and albuminocytological dissociation in CSF elevated the diagnostic certainty from “possible focal CIDP” to “focal CIDP” according to revised EFNS criteria, 2021. Steroid tapering was planned because of improvement.

However, he again presented with a right foot drop two months after discharge. This time he gave a clear history that foot drop was preceded by a prolonged period of crossed leg sitting on a chair. On examination, there was normal bulk and tone of all four limbs. The power of right and left foot dorsiflexion were 2/5 and 4/5 respectively. Bilateral foot evertors had 4/5 power. The rest of the muscle groups had 5/5 power. There was decreased sensation over the dorsum of the right foot in the superficial peroneal nerve distribution. Bilateral ankle and knee reflexes were absent. Jerks of the upper limb were within normal limits. Cranial nerves and cerebellar examination were within normal limits. There was no past or family history of a similar illness. NCS examination showed a conduction block in right and left peroneal nerves. Considering the very high likelihood of HNPP diagnosis because of the typical history and lower cost of targeted genetic analysis compared to whole exome sequencing, a targeted gene analysis for the *PMP22* gene was sent which showed a heterozygous deletion, confirming the diagnosis of HNPP. Steroids were tapered over the next six weeks and stopped. The patient was given genetic counseling and asked to avoid situations like bending on elbows, prolonged cross-legged sitting, etc., which can precipitate further attacks. The patient is planned to be kept in strict follow-up with periodic clinical examinations and electrodiagnostic studies. Repeat NCS and EMG may show subclinical progression in form of increasing distal nerve latencies and features of denervation, respectively. 

## Discussion

When our case first presented in OPD with left foot drop, on first impression it seemed to be a result of worsening radiculopathy or a complication of a recent surgical procedure. However, L5 radiculopathy also involves foot plantar flexion and inversion which were spared in our case. Isolated left foot dorsiflexor involvement suggested the absence of widespread lesions of the lumbosacral plexus. Depressed ankle reflexes could be explained because of radiculopathy; however, depressed knee reflexes could not be explained at that time. The patient was advised NCS test but was lost to follow-up.

He again presented in OPD after two months with complaints of worsening left foot drop. The patient was admitted and NCS and CSF studies were ordered apart from other routine tests. Pathology in case of foot drop can lie in almost any part of the neuraxis (muscles, myoneural junction, nerves, lumbosacral plexus, radiculopathy, motor neuron disease, spinal cord, or brain). Peroneal neuropathy is a common cause of foot drop. Sciatic neuropathies with preferential involvement of peroneal fibers can also present similarly [5]. The presence of sensory loss excluded myopathy, myoneural junction abnormalities, and motor neuron disease. Normal strength in ankle plantar flexor and invertors were against foot drop secondary to L5 radiculopathy. Preserved power in other muscle groups and absence of widespread sensory loss was against lumbosacral plexus involvement. There are reports of foot drop secondary to central causes, but these cases are very rare to be considered in primary differential diagnosis [6]. Isolated weakness of left foot dorsiflexor and evertors with a sensory deficit in deep peroneal nerve territory localized the lesion to the left peroneal nerve. The patient’s NCV was suggestive of delayed latency in the right peroneal nerve and conduction block in the left peroneal nerve. Also, his bilateral superficial peroneal potentials were absent. These findings in a patient with a stepwise progressive course of left foot drop with ankle and knee areflexia (knee areflexia not explained by radiculopathy) were suggestive of possible focal CIDP according to revised EFNS criteria, 2021. The finding of albumin-cytological dissociation in CSF and improvement with steroid therapy increased his diagnostic certainty for CIDP from “possible focal CIDP” to “focal CIDP” [7].

However, after two months he again developed a right foot drop and was admitted again. This time he gave a clear history of precipitation of the event after prolonged sitting in a cross-legged position in the chair. On examination, there was decreased strength in the dorsiflexion of both feet (right- 2/5, left- 4/5). There was also decreased strength in bilateral evertors (power- 4/5). Power in all other muscle groups was 5/5. Bilateral knee and ankle reflexes were absent. Sensory examination showed decreased sensation in the dorsum of the right foot. According to examination new lesion seemed to localize to the right peroneal nerve. NCS showed a new conduction block in the right peroneal nerve. All in all conduction block in bilateral peroneal nerves, absent bilateral superficial peroneal nerve amplitude, and increased distal motor and sensory latencies in bilateral median nerve were consistent with electrodiagnostic findings described in the literature in HNPP cases [8]. Advances in genetics have made the use of nerve biopsies obsolete in a suspected case of HNPP. However, sometimes in cases with atypical presentations like chronic idiopathic polyneuropathy together with absent family history nerve biopsy showing typical tomaculous lesions can lead us to suspect HNPP [9].

With a positive result for the genetic test, our diagnosis was changed to Hereditary Neuropathy with Liability to Pressure Palsy in place of focal or atypical CIDP. HNPP is a rare condition as compared to some other acquired demyelinating conditions like CIDP, its prevalence being 7/100000 to 16/100000 in the population. It is commonly caused by the deletion of the *PMP22* gene on chromosome 17 and has autosomal dominance transmission with variable expressivity. About 20% of cases are caused by a de-novo mutation. The disease typically results in mononeuropathies because of nerve injury at entrapment sites or other susceptible locations. Other presentations include polyneuropathy like presentation mimicking CMT disease and mono neuritis multiplex pattern [4]. MADSAM is a close differential diagnosis with mononeuritis multiplex like presentation. History of recurrent mononeuropathies with improvement and positive family history favor diagnosis of HNPP over MADSAM. Though CIDP or its variants can present with a relapsing-remitting course, such a course should raise suspicion of an alternative diagnosis. Electrophysiologically conduction block at entrapment sites, the presence of sural and median nerve abnormalities (decreased conduction velocity and increased distal latencies) should raise suspicion of HNPP over MADSAM. However, carpal tunnel syndrome (CTS) is such a common disease that median nerve abnormalities can be mistakenly considered secondary to accompanying CTS rather than part of the same disease process. Sometimes additional tests like a CSF study (to look for albuminocytological dissociation) can provide further insight. However, in our case finding of albuminocytological dissociation because of mildly elevated CSF protein wrongly tilted the balance in favor of an atypical CIDP variant.

HNPP is precipitated by conditions like prolonged crossed-leg sitting, squatting, leaning on elbows, carrying heavy backpacks, etc. The parent responsible for gene transmission can be asymptomatic in about 20% of cases. Our case had no family history of similar illness which can be because of de-novo mutation or asymptomatic parents because of variable expressivity. History of predisposing conditions is not always present as in our case as events may be precipitated by unnoticed microtraumas [4].

Management is primarily directed toward occupational and physical therapy including the provision of transient bracing like ankle foot orthosis. Prevention of further attacks by avoiding positions like crossed leg sitting, squatting, leaning on elbows, and wearing heavy backpacks on shoulders is of utmost importance. The prognosis for recovery is good. However, sometimes residual deficits can be left which are usually mild [4]. Our case with proper counseling has not shown any further event of nerve palsy till six months of follow-up. Providing genetic counseling for the chances of children being affected is essential.

There were many caveats in the diagnosis of focal CIDP in the first place. Features like increased CSF protein (albuminocytological dissociation) can be present in several conditions like CSF flow block because of prolapsed intervertebral disc (PIVD), diabetes, stroke, seizure, subarachnoid hemorrhage (SAH), brain tumor, etc. In fact, the first report of albuminocytological dissociation was in a case of disc prolapse [10]. A large case series of CIDP patients has shown that CSF protein levels below 100 mg/dl has got low specificity for the diagnosis of CIDP and mentions that the diagnosis of atypical CIDP is often wrong [11]. In our case also the primary diagnosis was focal or atypical CIDP and elevated CSF protein was lower than 100 mg/dl (91.3 mg/dl). The patient was diabetic and had lumbosacral disc disease which could have resulted in increased CSF protein. The apparent response to steroids can be because of the spontaneous improvement of reversible conduction block in HNPP. The revised EFNS CIDP guidelines also point to the fact that supportive criteria like elevated CSF protein and response to steroids can be non-specific and should be used cautiously [7]. In a series of 58 patients with L5 monoradiculopathy, 19 patients were found to have abnormal knee reflexes which were thought to be because of impairment of the proprioceptive drive from the pretibial muscles to spinal premotor excitatory interneurons contacting quadriceps motor neurons [12]. Decreased knee reflex in our case could have a similar basis. A similar confusion of HNPP cases with CIDP has been described in case series of 12 patients of HNPP, with three of them initially diagnosed as CIDP [13]. Shields et al. also reported one patient with foot drop initially diagnosed as CIDP and treated with intravenous immunoglobulin (IVIg) without benefit. A positive family history of polyneuropathy, past history of foot drop with improvement two years back, and paying attention to details of NCS results (diffusely absent SNAPs and increased distal motor latencies in upper and lower limbs) suggested a diagnosis of HNPP which was later confirmed by genetic studies [14]. In our case, there was no past or family history of similar illness, but bilateral median motor and sensory latencies were increased which was initially attributed to accompanying CTS. Recurrence of foot drop with cross-legged sitting and positive genetic test led to the diagnosis of HNPP in our case. Some cases have even shown an overlap of CIDP/HNPP phenotype causing further confusion [15]. There are also reports of CIDP being precipitated by recurrent episodes of demyelination secondary to hereditary neuropathies [16]. We also cannot rule out for sure whether our case was having isolated HNPP or was having an overlap of CIDP/HNPP because of widespread areflexia. A long-term follow-up is required to rule out the possibility of the HNPP/CIDP overlap phenotype. Currently, the patient is off steroids and doing well.

## Conclusions

Our case showed that diagnosis of atypical CIDP requires particularly close follow-up and observation as many times the final diagnosis turns out to be something else (HNPP in our case). Over-relying on mildly elevated CSF protein can lead to erroneous diagnosis as it can be a non-specific finding in patients with comorbidities. Apparent glucocorticoid response can be seen in hereditary conditions like HNPP because of reversible conduction block and is not specific for acquired conditions like CIDP. Knee jerk hyporeflexia or areflexia which caused confusion in our case leading to a diagnosis of CIDP can also be found in L5 radiculopathy because of impairment of the proprioceptive drive from the pretibial muscles to spinal premotor excitatory interneurons contacting quadriceps motor neurons. Furthermore, we cannot completely rule out HNPP/CIDP overlap phenotype in our case and a close follow-up is required.

## Appendices

**Table 1:** Investigations TLC: total leukocyte count; AST: serum aspartate aminotransferase; ALT: alanine aminotransferase; CSF: cerebrospinal fluid; TSH: thyroid-stimulating hormone; CCP: cyclic citrullinated peptides; NCS: nerve conduction study; LS: lumbosacral spine

	First admission	Second admission
Hemoglobin	15.3 g/dl	16.1 g/dl
TLC	6300/cumm	6000/cumm
Platelets	277000/cumm	219000/cumm
AST	22.4 IU/L	28.4 IU/L
ALT	19.5 IU/L	16.3 IU/L
Sr. Bilirubin (direct/indirect)	0.39/0.25 mg/dl	0.38/0.13 mg/dl
Sr. Creatinine	1.09 mg/dl	1.12 mg/dl
Sodium	138 meq/l	141 meq/l
Potassium	4.1 meq/l	5.1 meq/l
CSF cells	6/cumm (lymphocyte- 100%)	NA
CSF protein	93.1 mg/dl	NA
CSF glucose	81 mg/dl	NA
Vitamin B12	295 picogram/ml	NA
TSH	3.45 micro IU/ml	NA
RA factor/anti CCP	Negative	NA
ANA (antinuclear antibodies)	Negative	NA
HIV/HBSAg/HCV	Negative	NA
Serum protein electrophoresis	Negative	NA
Urinary protein electrophoresis	Negative	NA
Urinary BJ protein	Negative	NA
NCS	Conduction block in left peroneal nerve	Conduction block in bilateral peroneal nerve
MRI LS spine (before surgery)	Right-sided spondylolysis with grade 1 anterolisthesis of L5 over S1. Asymmetric broad-based disc bulge at L5-S1 indenting the thecal sac	
